# Production of rGO-Based Electrospinning Nanocomposites Incorporated in Recycled PET as an Alternative Dry Electrode

**DOI:** 10.3390/polym14204288

**Published:** 2022-10-12

**Authors:** Michelle Chizzolini Barbosa, Claudia do Amaral Razzino, Thiago Domingues Stocco, Moisés das Virgens Santana, Anupama Ghosh, Luiz Fernando Pereira, Carlos Julio Tierra-Criollo, Anderson Oliveira Lobo

**Affiliations:** 1 Research and Development Institute, University of Vale do Paraiba—UNIVAP, São Jose dos Campos 12244-000, SP, Brazil; mi.chizzolinib@gmail.com (M.C.B.); claudiarazzino@gmail.com (C.d.A.R.); 2 Bioengineering Program, Scientific and Technological Institute, Brasil University, São Paulo 08230-030, SP, Brazil; thiago.stocco@ub.edu.br; 3 Interdisciplinary Laboratory for Advanced Materials, Materials Science and Engineering Graduate Program, Federal University of Piaui, Teresina 64049-550, PI, Brazil; moisesdvs@outlook.com; 4 Department of Chemical and Materials Engineering—DEQM, Pontifical Catholic University of Rio de Janeiro, Rio de Janeiro 22453-900, RJ, Brazil; anupama1984@gmail.com; 5 Biomedical Engineering Program-PEB, Federal University of Rio de Janeiro, Rio de Janeiro 21941-914, RJ, Brazil; f.pereiraluiz@peb.ufrj.br

**Keywords:** recycled polyethylene terephthalate, reduced graphene oxide, electrospinning, dry electrodes, current perception threshold, sinusoidal electrical stimulation

## Abstract

In this work, Coca-Cola^®^ bottles were reused as a PET polymer (rPET) source to produce electrospun polymeric nanofibers. The nanofibers were electrospun from polymer solutions with different concentrations of reduced graphene oxide (rGO) incorporated for applications in somatosensory electrical stimulation. The rPET/rGO nanofiber mats were characterized by SEM, TEM, Raman, DSC, TGA, and DMA and the results showed that the incorporation of rGO in electrospun rPET fibers produced rPET/rGO composites. The rPET/rGO composites were then evaluated for possible application as dry electrodes. Moreover, with a preliminary test of numerous volunteers, the rPET/rGO dry electrode showed promising results. The rPET/rGO electrodes showed good performance and applicability to make dry electrodes, and these have applications as dry or wearable electrodes to produce electrochemical sensors.

## 1. Introduction

Somatosensory electrical stimulation is referred to as a method of activating the excitable tissue of the human body through the application of an electrical current. Electrical stimulation applied at the sensory or motor threshold is suggested to modulate neuronal activity. This has shown potential clinical applications such as pain relief control of epileptic seizures [1], muscle activation [2,3], relief of symptoms of Parkinson’s disease [4], improvement of motor function after a stroke [5,6], and spasticity control [7], as well as the diagnosis of neural injuries [8].

Among the range of techniques used in this approach is transcutaneous electrical stimulation, in which an electrical current (especially a sinusoidal electrical current) is applied non-invasively through electrodes positioned on the skin surface [6,9]. In this context, the characteristics and quality of the electrodes are essential for electrostimulation and for the acquisition of bioelectrical signals, since they are responsible for the interface between the human body and the electrical circuit [10].

The most used surface electrodes are currently self-adhesive Silver/Silver Chloride (Ag/AgCl) electrodes. Despite their numerous advantages, as compared to other surface electrodes (e.g., conductive rubber and stainless-steel electrodes), Ag/AgCl electrodes have some inconveniences, such as causing irritability and long-term skin burns, sensitivity to electromagnetic interference, noise in the system due to dehydration, need to use conductive gels or creams, and therefore, greater prior preparation in addition to the need to clean the skin after application [11,12,13,14,15]. To overcome these difficulties, flexible dry electrodes have been considered to be a good alternative to replace dense matrix electrodes. The reason is that they could improve signal interference and reduce skin damage by continuous contact and dispense with the use of gels that may cause allergies [12,16,17].

The application of nanomaterial-based dry electrodes has recently received attention as a substitute for traditional Ag/AgCl electrodes due to its unique properties, such as high surface area, excellent electrical conductivity, and good flexibility, in addition to being able to be used in more complex capture systems of electrical signals [18,19,20].

Furthermore, a low-cost form of flexible dry electrode production can be purchased using polymer matrix-based nanocomposites. The use of recycled PET (rPET) fibers is a strong candidate, along with nanometric form, to overcome problems such as flexibility, adhesion, biocompatibility, and noise reduction [21]. Other than an alternative to maximize the exploitation of municipal solid waste, it also produces a new device with favorable properties and prices [22].

The challenge of using rPET substrates is the insertion of conductivity into the polymeric matrices. As an insulating polymer, it needs excellent conductive materials as reinforcement and materials that can have good incorporation into the polymer, such as mica, graphene, and carbon nanotube [23]. Carbon materials, such as graphene and carbon nanotubes (CNTs), are suitable additives that are able to be evenly distributed within polymer matrices, thereby increasing the material resistance and introducing conductivity to the nanocomposite [24,25].

The abundance of available graphene makes it a more attractive option than CNTs for large-scale production, in addition to having a lower cost [26]. The graphene reduction method, in turn, has been a necessary precursor to producing large quantities of a type of graphene, generally called reduced graphene oxide (rGO) [27]. This carbon-based nanomaterial can be used as a flexible and biocompatible substrate due to its high thermal, chemical, mechanical, and electrical properties [28,29].

Dry electrodes involving rGO have shown effective performance, high flexibility, and satisfactory usability, and therefore, the potential for application in somatosensory electrical stimulation [30,31,32]. Thus, in this work, we developed a new nanostructured device to be used as a flexible dry electrode-based rGO embedded in electrospun rPET fibers (rPET/rGO). We aim to produce an efficient, sustainable, and low-cost surface electrode for application in the transcutaneous electrical stimulation technique.

## 2. Materials and Methods

### 2.1. Reagents

Reduced graphene oxide (rGO, 98%, Cod: HP-RGO-025G) was purchased commercially from Graphene Supermarket. Trifluoroacetic acid (TFA 99%) and dichloromethane (DCM 99.5%) were purchased from Sigma-Aldrich and Synth, respectively, and used as solvents (TFA/DCM 80:20 *v*/*v*) for the electrospinning of rPET and rPET-rGO. All the bottles used as the PET source were from Coca-Cola^®^ (from The Coca-Cola Company, Atlanta, GA, USA). All other reagents were of analytical grade and were used as received.

### 2.2. Preparation of the rPET Solution for Electrospinning

The bottles were cleaned with household detergent, sodium hypochlorite solution, and ultrapure water. Detergent and sodium hypochlorite were used to eliminate possible contaminants of this type, and ultrapure water was used to remove traces of the solvents used. A similar PET cleaning procedure was performed by Mendiburu–Valor et al. [33]. After that, one was dried and cut into pieces to prepare the rPET electrospinnable solutions. The rPET electrospinnable solution at different concentrations 15–30% (*w*/*v*) was prepared in a blend of TFA/DCM 80:20 (*v*/*v*) according to the work by Veleirinho et. al. [34,35]. First, 15–30 g of rPET was solubilized in 80 mL of TFA. After that, 20 mL of DCM was added to the mixture of the rPET and TFA. This solution was kept under magnetic stirring at 1200 rpm and at room temperature for 24 h.

### 2.3. Preparation of the rPET-rGO Solution for Electrospinning

The rPET-rGO electrospinnable solution of the rPET concentration of 15% (*w*/*v*) and with different concentrations of rGO (0–1.25 %, *w*/*v*) were prepared in a blend of TFA/DCM 80:20 (*v*/*v*). First, different quantities of rGO were added to 80 mL of TFA. This suspension was kept under ultrasonic stirring for 1 h. Thereafter, 20 mL of DCM and 15 g of rPET were added to the suspension of rGO and TFA. This solution was kept under magnetic stirring at 1200 rpm and at room temperature for 24 h.

### 2.4. Electrospinning Process

The electrospinnable solutions were placed into a 10 mL glass syringe with a metallic needle with an inner diameter of 18 G (1.3 mm). The solution flow rate was controlled by a syringe pump (NE-300 Just Infusion™). A high-voltage power supply (Spell-man/Bertan 230) was employed to generate the electric field. All electrospinning process parameters were evaluated according to Table 1, with a flow rate of 0.5 mL/min to ensure ideal conditions. In electrospinning, the viscosity of the solution, the distance from the needle to the collector and the voltage influenced the formation of fibers, and outside the ideal conditions for electrospinning, no fiber formation occurred. Therefore, the parameters of the had to be evidenced to facilitate the reproducibility of the work. The intervals in Table 1 are used to infer the best electrospinning parameters for a particular type of polymer and equipment used. The optimal parameters can be selected by characterizing the viscosity as well as the fibers, or they can be selected by visual analysis of the fiber formation. Fibers are generally only formed with these ideal parameters.

All the experiments were carried out in air at room conditions (temperature: 25 ± 2 °C and humidity 40 ± 5%) and a collection time of 1 h. Graphene oxide is a material that can diffuse into PET and provide electrical properties to the final product, even in small concentrations. Thus, rPET electrospun nanofibers mats with different concentrations of rGO incorporated were produced and are denominated according to Table 2.

### 2.5. Characterization of the Electrospun Nanofibers Mats

Morphology and diameter of the electrospun nanofibers mats and the incorporation of rGO at different concentrations into the rPET electrospun nanofibers were analyzed by Scanning Electron Microscopy (SEM—Zeiss EVO MA10) and Transmission Electron Microscopy (TEM—FEI Tecnai G2F20). The SEM analysis was performed at an accelerating voltage of 20 kV. The average fiber diameter was calculated from 50 measurements conducted on each electrospun nanofiber mat using the SEM images with a magnification of 5000 and the software Image J (version 1.37c, Wayne Rasband, National Institute of Health, Bethesda, MD, USA).

Differential Scanning Calorimetry (DSC—TA Instruments DSC Q20) and ThermoGravimetric Analysis (TGA—NETZSCH STA 449F3) were used to study the thermal properties of the electrospun nanofibers mats. DSC thermograms were obtained using samples from the electrospun nanofibers mats of 7–10 mg into sealed aluminum pans, heated from 0 to 300 °C at a heating rate of 10 °C/min, under a constant flow of dry nitrogen. With the DSC thermograms, it was possible to obtain the glass transition temperature, temperatures, and enthalpies of melting and crystallization, and to determine the degree of crystallinity (χ*_c_*) that was calculated from Equation (1) [36]:

(1) $$\chi_{c}\;\left(\%\right)=\frac{\Delta H_{f}-\Delta H_{c}}{\Delta H_{f}^{0}}\times 100$$

where $\Delta H_{f}$ is the melting enthalpy, $\Delta H_{c}$ is the enthalpy of crystallization and $\Delta H_{f}^{0}$ is the heat of the fusion of the completely crystalline PET (135.8 J/g) [37].

TGA was used to study the thermal properties of the electrospun nanofibers mats, namely the decomposition temperature and thermal stability. Samples of 10–12 mg were submitted to heating cycles of 30 to 900 °C, with a rate of 20 °C/min.

Tensile tests (DMA—TA Instruments Q800) were performed at 25 °C using the tension mode. The samples (5.0 × 6.4 × 0.2 mm, length × width × thickness) were strained to 18 N or to a failure at a constant rate of 1 N·min^−1^. These tests were carried out with three specimens from each sample of the electrospun nanofiber mats.

Raman spectroscopy (Bruker Senterra coupled to an Olympus BX50 microscope and a charge coupling device (CCD) as a detector) analysis was performed to investigate the possible structural alterations on the rPET/rGO electrospun nanofibers mats. The Raman spectra were recorded at room temperature. The Raman spectrometer was equipped with 532 nm (2.33 eV) and 785 nm (1.56 eV) wavelengths and 5 mW and 10 mW excitation light sources, respectively. The time and cycle of spectral accumulation were varied to reach the maximum signal-to-noise ratio. The spectrometer was adjusted to obtain a scanning spectral resolution of 3 cm^−1^ in the spectral range of 100 to 3700 cm^−1^.

### 2.6. rPET/rGO Electrospun Nanofibers Mats as a Substrate for Dry Electrodes

#### Preparation of rPET/rGO Electrodes

**Figure 1:**
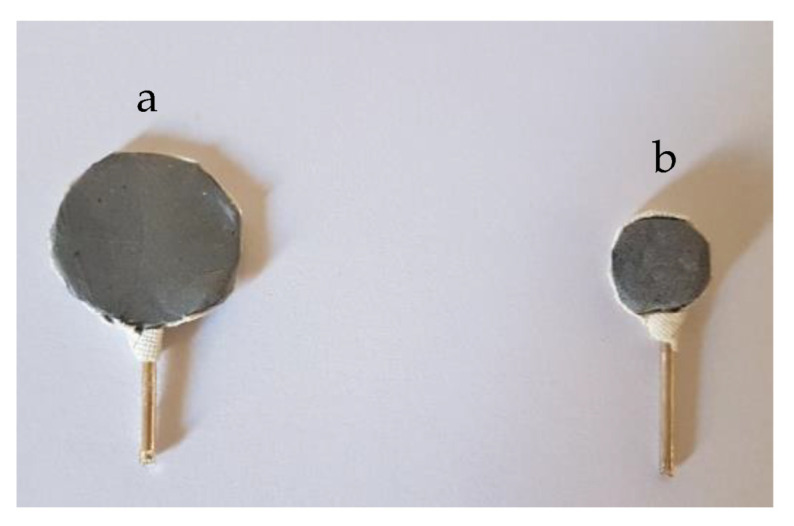
rPET/rGO-1.25 electrodes of (**a**) 18 mm diameter and (**b**) 10 mm diameter.

For the preparation of the rPET/rGO electrodes, PET holders of 18- and 10-mm diameter were coated with rPET/rGO electrospun nanofibers mats. Platinum wires were used as an electrical contact, as shown in Figure 1.

### 2.7. Test of the Sensory Perception Threshold (ST) for Sinusoidal Current Stimuli

This test was approved by the Ethics Committee of the Federal University of Rio de Janeiro (UFRJ) and registered under protocol CAAE 44944515.4.0000.5257. The volunteers were instructed about the procedures to be performed and were only included in the study after signing up the “Free and Clarified” Consent Term. The procedures were performed in a controlled environment (25 ± 2 °C) at the Laboratory of Image and Signal Processing (LAPIS) at the UFRJ. The NeuroStim System [38] was used for electrical stimulation with frequencies of 5 and 3000 Hz. The system can generate a programmable electrical current stimulus as high as 8 mA, with a resolution of 8 μA. The waveforms may range from 1 Hz to 5000 Hz at steps of 0.1 Hz, with a total harmonic distortion (THD) below 1.5%. The study included 10 subjects, aged 20 to 36 years old, without cognitive impairment, after a minimal mental health examination, and none used any medicine that interferes with the nervous system. Volunteers were positioned in a comfortable armchair with their upper limbs supported by the chair arms. The stimulation was applied on the median nerve, with the electrodes positioned in the wrist region, in both upper limbs. Firstly, the region was cleaned with alcohol-embedded cotton, and then the stimulation was applied using two 10 mm diameter Ag/AgCl electrodes with a thin amount of conductive gel, separated by 2 cm between centers. The same procedure and configuration were used for the rPET/rGO-1.0 dry electrodes (10- and 18-mm diameter, without conductive gel). Subjects were instructed to remain relaxed, with their eyes open, during all the procedure and to press a button positioned in their dominant hand whenever they felt any somatosensory perception (stinging, heat, needling, pressure, contraction, vibration, tingling, and/or itching). Such information was used to determine ST. The protocol described by Martins et al. [38] was used to identify ST. In this work, two frequencies (*f*) of 5 and 3000 Hz were used. The initial amplitude (inA) of 100 and 800 µA were used for the stimuli of 5 and 3000 Hz, respectively. The initial increment (inI) was 50 and 100 μA for 5 and 3000 Hz frequencies, respectively. The stimulation time (STt) and resting time between stimuli (Rt) were 3 s for both frequencies. The parameters are shown in Table 3.

Due to time constraints and the availability of volunteers, the rPET/rGO-1.0 electrode was randomly chosen for the sensory threshold (ST) test for sinusoidal current stimuli. During the experiment, the amplitude increased until the perception of stimulation by the volunteer was possible. Once the perception was obtained, the analysis was interrupted, and the value of the threshold indicated in uA was recorded. This value was used to determine the cognitive-motor response to the stimulus perception and was compared to the values of the thresholds obtained by the Ag/AgCl electrodes and the rPET/rGO-1.0 electrodes (Figure 1).

## 3. Results and Discussion

### 3.1. Microstructure (SEM and TEM Results)

Morphology and diameter of the electrospun nanofibers mats and the incorporation of rGO at different concentrations into the rPET electrospun nanofibers were analyzed by scanning electron microscopy (SEM) and transmission electron microscopy (TEM).

**Figure 2:**
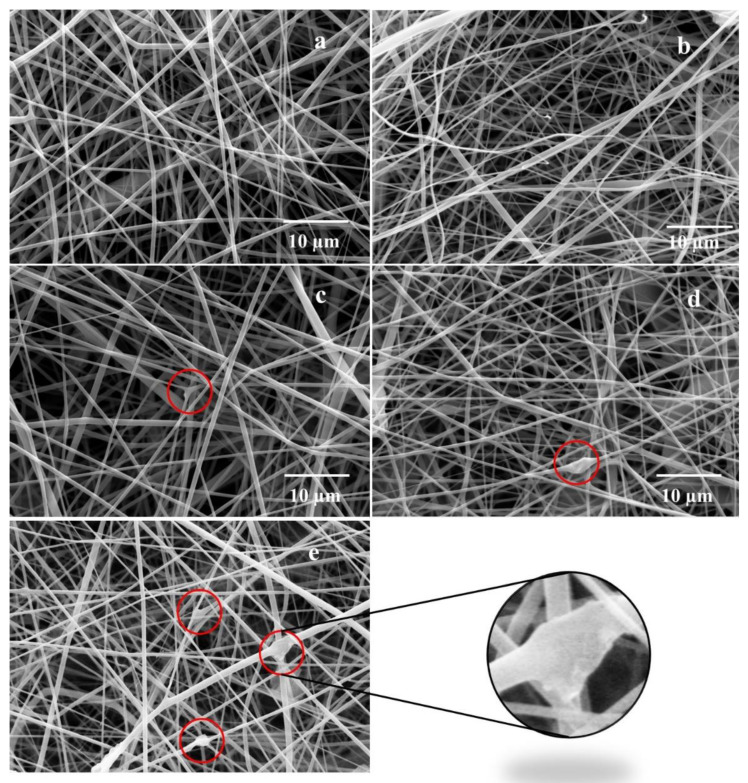
SEM images of (**a**) rPET; (**b**) rPET/rGO-0.5; (**c**) rPET/rGO-0.75; (**d**) rPET/rGO-1.0; and (**e**) rPET/rGO-1.25.

Figure 2 shows the SEM images of rPET and rPET/rGO polymer nanofibers mats, where fine fibers with a random distribution, smooth and bead-free, are observed. With the increased concentration of rGO in the polymeric solution, the nonuniformity of the fibers and the presence of agglomerates (red circles) in their structure can be observed.

**Figure 3:**
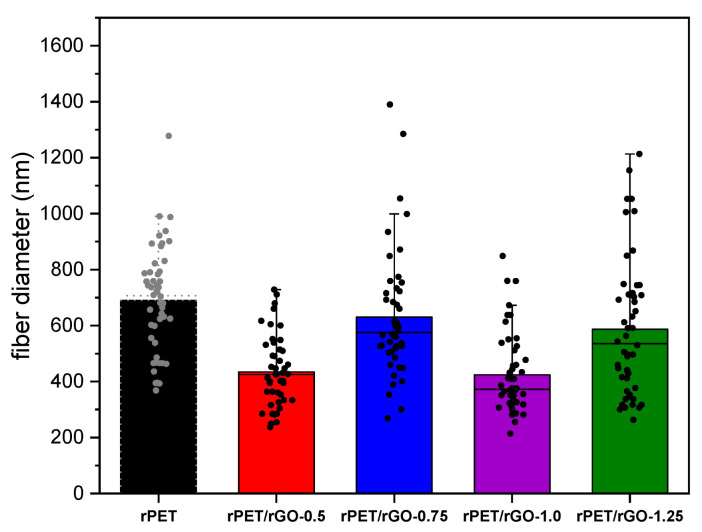
The mean fiber diameter of rPET and rPET/rGO nanofibers.

The mean diameter of these polymer nanofibers was presented in Figure 3, and no correlation was observed between the mean diameter and the concentration of rGO.

According to Bhardwaj and Kundu [39], an increase in the polymeric solution conductivity may lead to a significant decrease in the diameter of electrospinning fibers due to the solution stretching during the electrospinning process and the greater electric charges mobility in the polymeric solution. However, for rPET/rGO fibers, there was no linear relationship between the relative fiber diameter and the concentration of rGO in the polymer solution. This can be explained by the non-homogeneity of the solution, as confirmed by the agglomerations observed on the SEM images.

**Figure 4:**
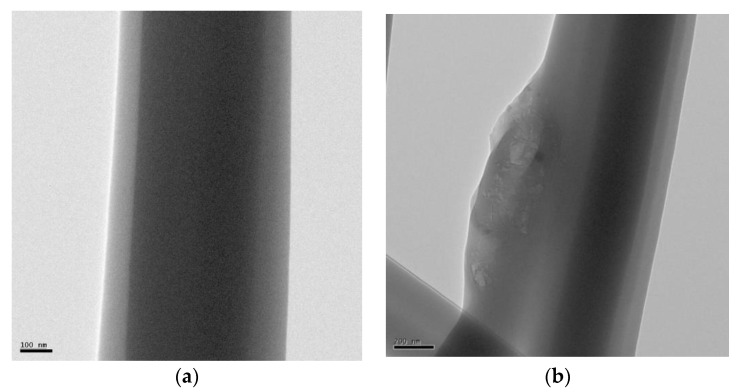

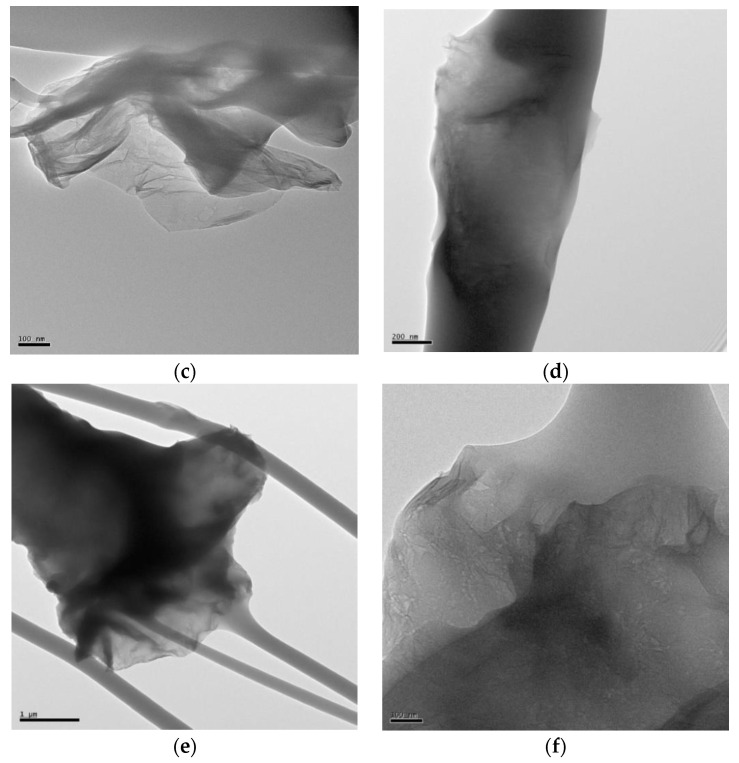
Transmission Electron Microscopy of (**a**) rPET; (**b**) rPET/rGO-0.5; (**c**) rPET/rGO-0.75; (**d**) rPET/rGO-1.0; and (**e**,**f**) rPET/rGO-1.25.

Figure 4 shows the TEM images of rPET and rPET/rGO polymer nanofibers with different rGO concentrations, and the presence of rGO can be observed in all rPet/rGO samples. The graphene sheets are sometimes exposed, which is critical to confer electrical conductivity to the rPET/rGO polymeric nanofiber mats [40].

### 3.2. Raman Spectroscopy Results

**Figure 5:**
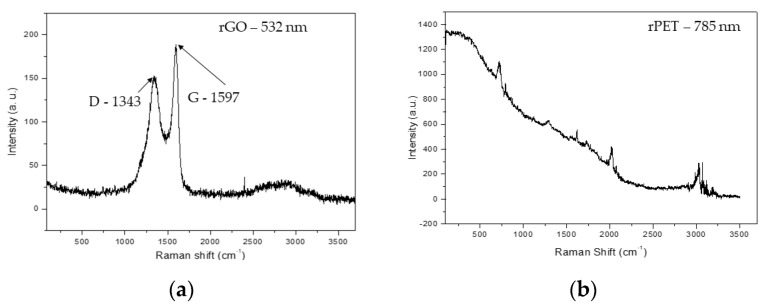
Raman spectra of (**a**) rGO and (**b**) rPET samples.

Raman spectroscopy analysis was performed to investigate the possible structural alterations on the rPET/rGO electrospun nanofibers mats. Figure 5 shows the Raman spectra of rGO and rPET as a reference for the structural analysis of the rPET/rGO composites. In Figure 5a, the D (1343 cm^−1^) and G (1597 cm^−1^) bands characteristic of graphene are observed [41]. Whereas for the rPET samples, high luminescence was observed at the 532 nm laser. With this, the analysis of the rPET peaks was suitable for a 785 nm laser, as observed in Figure 5b.

**Figure 6:**
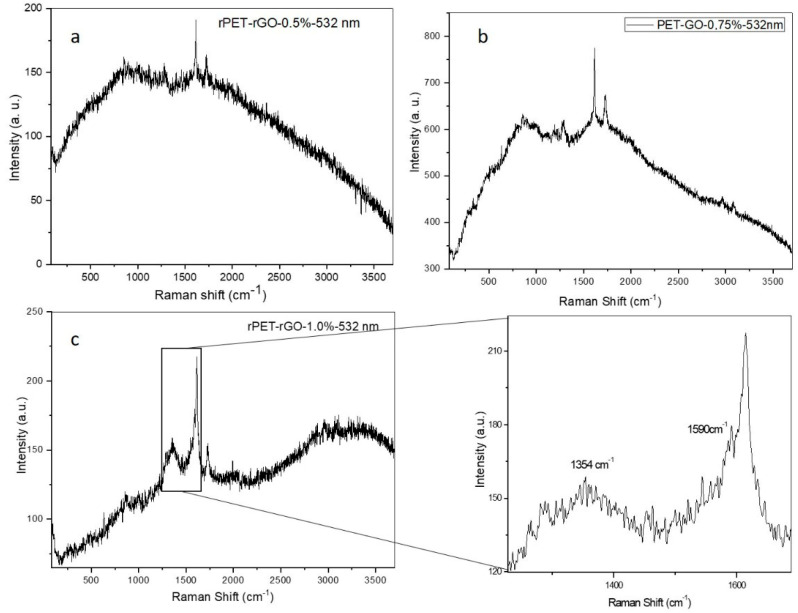
Raman spectra of (**a**) rPET/rGO-0.5; (**b**) rPET/rGO-0.75; and (**c**) rPET/rGO-1.0 composites, with an emphasis on the D and G bands, characteristic of graphene.

In Figure 6a,b, it is observed that, in the rPET/rGO composites with rGO concentrations of 0.5% and 0.75% (*w*/*v*), no peaks referring to the presence of rGO are identified. At higher rGO concentrations, as shown in Figure 6a,c, the D and G bands began to appear as a shoulder of the 1614 cm^−1^ band.

**Figure 7:**
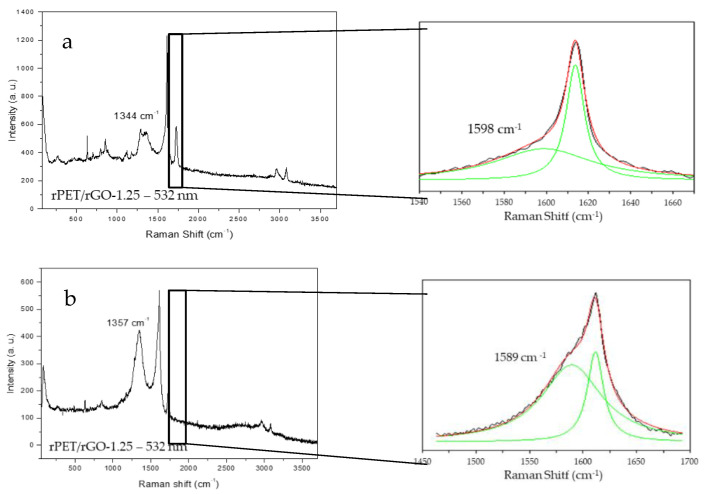
Raman spectra of the rPET/rGO-1.25 samples at the shifts of (**a**) 1598 cm^−1^ and (**b**) 1589 cm^−1^, characteristic of graphene, at different intensities.

Figure 7 shows the different spectra of the rPET/rGO-1.25 composite sample where we can observe the D and G bands. For the same sample, peaks of different intensities are observed, with a lower intensity (Figure 7a) and higher intensity (Figure 7b), depending on the position of the sample on the analysis support.

rPET/rGO-0.5 and rPET/rGO-0.75 did not show characteristic graphene peaks in the RAMAN analysis, but the incorporation of rGO in the respective samples was evidenced by TEM images. In conclusion, although the incorporation of rGO occurred in lower concentrations, these were insufficient for evaluation using the Raman technique. For the rPET/rGO-1.25 composites, distinct rGO peak intensities were observed, thus indicating a lack of microscopic homogeneity, which was also confirmed by the SEM and TEM techniques.

### 3.3. Thermal Behavior, Degradation, and Mechanical Properties

#### 3.3.1. Differential Scanning Calorimetry (DSC)

**Figure 8:**
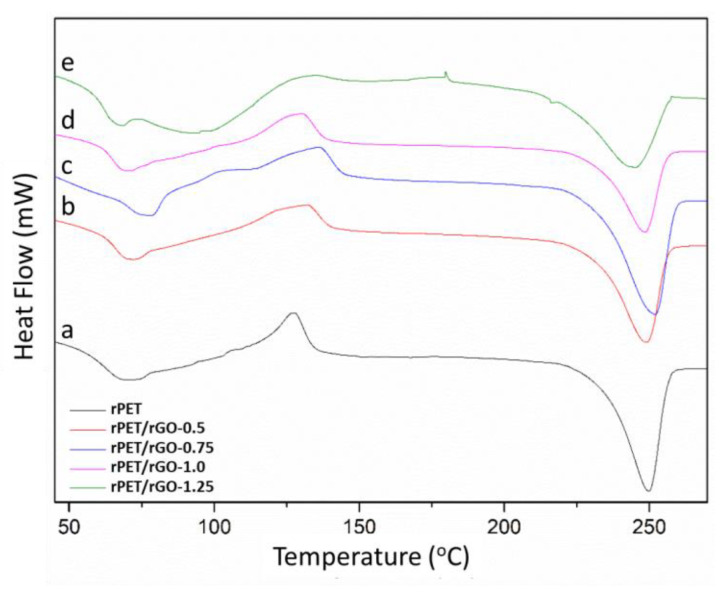
DSC curves of (**a**) rPET; (**b**) rPET/rGO-0.5; (**c**) rPET/rGO-0.75; (**d**) rPET/rGO-1.0; and (**e**) rPET/rGO-1.25.

The DSC curve (Figure 8) is typical of those expected for PET. It shows glass transition, an exothermic crystallization peak, and well-defined endothermic melting peaks [34]. In the DSC curves, the glass transition of rPET/rGO composites, crystallization temperature (Tc), and melting temperature (Tm) can be observed, as well as their respective enthalpies used to calculate the degree of crystallinity.

Table 4 shows that, with an increasing rGO concentration incorporated into the fibers, there was a slight increase (5% to 13%) in the glass the transition of rPET/rGO-0.5, rPET/rGO-0.75, and rPET/rGO-1.0 samples. For the rPET/rGO-1.25 sample, the Tg value is very similar to that of rPET. Regarding the degree of crystallinity, the values vary without correlation with the rGO concentration, decreasing for rPET/rGO-0.5, 1.0, and 1.25 samples, and increasing for rPET/rGO-0.75 relative to rPET.

The effect of cold crystallization is present in primarily amorphous materials, with a reorganization of their chains during heating. In general, Tg changes above 20°C are uncommon with the addition of graphene particles, and in most cases, are reported values below 10 °C. CHENG et al. [42] had an increase of 5% in Tg on polyvinyl alcohol samples electro-fused with rGO at a concentration of 1% (*w*/*v*). The increase in Tg of the composites can be attributed to the confinement effect of the graphene sheets in the polymer matrix chains [43].

According to the data in Table 4, the crystallinity of the rPET fiber is similar to that of commercial PET fibers reported by Veleirinho et al. [34]. The research also reported that the degree of crystallinity of the raw commercial PET grains is 15%, while the electrospun PET materials presented crystallinity in the range of 21% to 23% [34].

The addition of nanomaterials to polymers causes an increase in their crystallinity rate, as the nanomaterial acts as a nucleating agent. This nucleation effect is significantly enhanced when homogeneous dispersion of the nanomaterial in the composite occurs [44]. The decrease in crystallinity of the rPET/rGO samples can be justified by the presence of rGO, whose sheets act as heterogeneous nucleation points, decreasing the free volume and creating constraints on the polymer chains, thus interfering in the reorganization and formation of crystals [45].

Low crystallinity is desired in materials intended for flexible electrodes. Materials with higher crystallinity are harder and more thermally stable, but more brittle. Composites with lower crystallinity, like those obtained in this work, even with the incorporation of rGO, have properties of amorphous structures, providing elasticity and impact resistance.

#### 3.3.2. Thermogravimetry Analysis (TGA)

**Figure 9:**
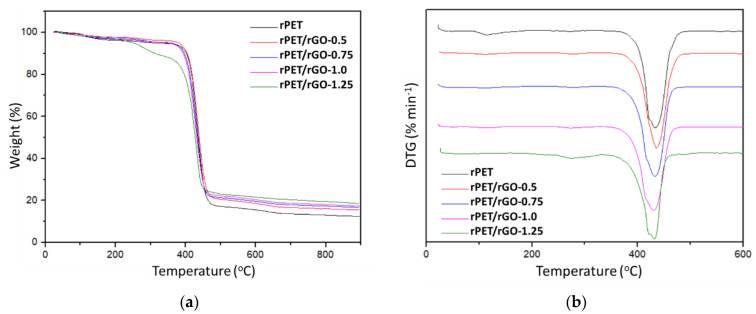
(**a**) TGA and (**b**) DTA curves of rPET and rPET/rGO polymer nanofibers at a heating rate of 20°C in N_2_.

Figure 9 displays the thermograms for the rPET and rPET/rGO samples, and the obtained data are shown in Table 5. It is observed that the thermal decomposition of the rPET and rPET/rGO-0.5, 0.75, and 1.0 samples occur in a single step, indicating the materials’ effective compatibility in the composite. The rPET/rGO-1.25 sample undergoes thermal decomposition in two steps. The first stage, from 220°C to 325°C, can be attributed to eliminating oxygen-containing compounds and oxide groups from the graphene sheets [46]. The second stage, approximately 350 to 460°C, which is the degradation of the polymer, was maintained at a similar temperature as the rPET samples.

In Table 5, looking at the degradation temperatures and peak temperatures (DTG) for the rPET and rPET/rGO samples, there are no significant differences between the temperature values. However, the rPET/rGO-0.5 sample shows higher thermal stability than the others. This can be explained because smaller amounts of rGO can be more effectively incorporated into the polymeric matrices, thus contributing to the thermal stability of the composite. Furthermore, the residual mass value increases with the increase in the amount of rGO in the composites. This can be explained by the high thermal stability of the graphene sheets.

#### 3.3.3. Mechanical Properties—Tensile Testing

For the tension tests, three specimens of each rPET and rPET/rGO composite sample were used, and the graphics (data not shown) presented significant differences between the scans of the same sample. This can be explained by the heterogeneity of the rGO distribution in each sample. Table 6 shows the average values of tensile strength, elongation, and Young’s modulus of rPET and rPET/rGO electrospun fiber mats, where it is observed that the samples with an rGO concentration of 0.5% (*m*/*v*) (rPET/rGO-0.5) showed a tensile strength similar to the rPET samples, with a reduction of only 6.8%. For the rPET/rGO-1.25 samples, the reduction in tensile strength was 58.6%. There was also a decrease in the elongation values and Young’s modulus with the increased concentration of rGO in the samples.

Graphene dispersion in polymer matrices, according to Wakabayashi et al. [47], causes graphene sheets to adopt wavy/wrinkled structures that can reduce the Young’s Modulus values, as the crumpled structures tend to inflate rather than stretch when stress is applied. In addition, the mechanical properties of the composites can be altered by problems of interfacial adhesion and/or spatial distribution of the graphene in the polymer matrix [45].

### 3.4. Test of the Sensory Perception Threshold (ST) for Sinusoidal Current Stimuli

**Figure 10:**
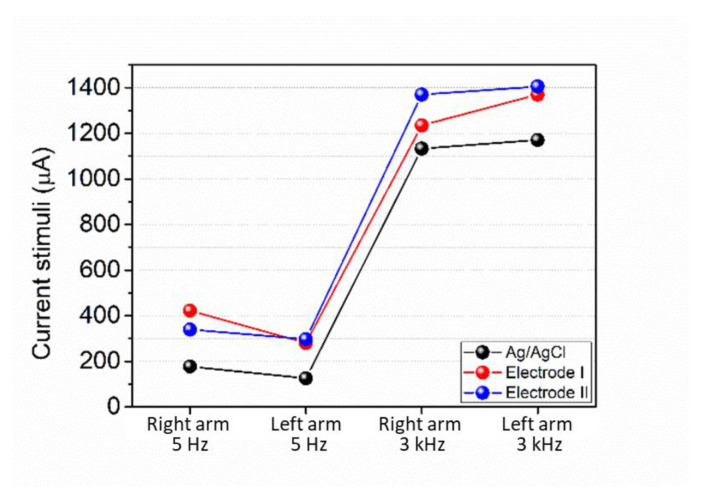
Graph the current stimuli values Ag/AgCl and rPET/rGO-1.0 electrodes, as a function of the stimulated limb, at frequencies of 5 Hz and 3000 Hz.

The applicability of the rPET/rGO electrospun nanofibers mats as a substrate for dry electrodes was evaluated by the sensory perception threshold (ST) test for sinusoidal current stimuli. The ST corresponds to the lowest current intensity capable of eliciting perception [38]. Figure 10 shows the graph of the mean sensitivity threshold values, in µA, for Ag/AgCl and rPET/rGO-1.0 electrodes, as a function of the stimulated limb, at frequencies of 5 Hz and 3000 Hz (Table 7). In addition, we observed higher threshold values for rPET/rGO electrodes. Between rPET/rGO-1.0 electrodes (ϕ 18 mm), called electrode I, and rPET/rGO-1.0 electrodes (ϕ 10 mm), called electrode II, we observed that the electrode II and Ag/AgCl electrode have linear performance at different frequencies, with different current magnitude. According to Bohórquez et al. [48], the constitution of the electrode materials, the particularities of any body, as well as the diameter of the segment to be stimulated, are essential to the impedance variations of the system. In theory, the electrode size is inversely proportional to the current density, i.e., smaller electrodes cause the current to pass through a smaller area, obtaining lower impedances, thus improving the electrode-skin sensitivity.

The sensations produced by the stimulation of the sinusoidal current, for Ag/AgCl electrodes, at a frequency of 5 Hz were needling and tingling, while for the frequency of 3 kHz they vibration, contraction and tingling, the latter being the most evident. For electrode I, the more perceptible sensations were vibration, pricking, and tingling at 5 Hz. The sensations of vibration and tingling were reported at 3 kHz, like those produced by the Ag/AgCl electrodes. Electrodes II, at a frequency of 5 Hz, gave off vibration and tingling sensations, and at a frequency of 3 kHz, vibration and prickling sensations.

Studies indicate that low-frequency stimulations may be related to type C sensations (prick, needling, and heat) and high frequencies to type Aβ sensations (vibration, pressure, and contraction) [49]. It was observed that electrode I presented current stimuli values and produced sensations that were mostly similar to the conventional Ag/AgCl electrode for both high and low frequencies. In addition, electrode II produced C-type sensations at high frequencies.

## 4. Conclusions

Electrospun nanofiber mats from polymer solutions of rPET and rPET with rGO incorporated have been successfully electrospun. Characterization techniques show the formation of the fibers and the incorporation of rGO into them. However, they also show that, in many cases, there was no correlation between the variation in fiber properties and the concentration of rGO. This can be explained by the inhomogeneity of the rGO in the fibers. For future work, this should be improved by increasing the dispersion of rGO in the polymer solution before the electrospinning process, which can be achieved by sonication. The rPET/rGO nanofiber mats were used to make dry electrodes, and their performance was compared to that of conventional Ag/AgCl electrodes. Due to the time and number of volunteers available for the experiments, the electrode with 1.0% (*w*/*v*) of rGO incorporated into the fibers was randomly chosen. The rPET/rGO-1.0 electrode showed good performance and applicability to make dry electrodes. This preliminary study showed the potential application of rPET/rGO electrospinning mats as a material to produce flexible electrodes. These have applications as dry or wearable electrodes to produce electrochemical sensors, with a low concentration of rGO and recycled PET as the matrix, and are thus a low-cost product and one that is more applicable for recycled PET, in addition to having an added value.

## Figures and Tables

**Table 1:** Electrospinning process parameters.

Parameters	Experimental Range	Optimal
rPET solution concentration	15–30% (*w*/*v*)	15% (*w*/*v*)
Applied voltage	15–20 kV	15 kV
Needle-collector distance	10–15 cm	10 cm

**Table 2:** Concentrations of rGO incorporated into rPET electrospun nanofibers mats.

Electrospun Nanofibers Mats	Concentration of rGO (*w*/*v*)
rPET	0
rPET/rGO-0.5	0.5%
rPET/rGO-0.75	0.75%
rPET/rGO-1.0	1.0%
rPET/rGO-1.25	1.25%

**Table 3:** Parameters used in the identification of ST.

f (Hz)	inA (µA)	inI (µA)	STt (s)	Rt (s)
5	100	50	3	3
3000	800	100	3	3

**Table 4:** Results obtained from DSC thermograms. Tg (Glass Transition); Tc (Cold crystallization temperature); Tm (Melting temperature); ∆*Hc* (Cold crystallization enthalpy); ∆*Hf* (Enthalpy of fusion); and *Xc* (Degree of crystallinity).

Samples	Tg (°C)	Tc (°C)	Tm (°C)	∆*Hc* (J/g)	∆*Hf* (J/g)	*Xc* (%)
rPET	62.63	127.81	249.73	10.84	41.38	21.81
PET/rGO-0.5	65.77	132.07	249.14	9.55	37.56	20.11
PET/rGO-0.75	70.64	136.32	251.73	10.46	45.56	25.26
PET/rGO-1.0	70.77	130.13	249.01	9.862	33.37	16.96
PET/rGO-1.25	62.90	129.39	244.94	10.55	40.45	21.62

**Table 5:** Results obtained by TGA and DTA curves of the rPET and rPET/rGO samples.

Samples	Degradation Temperature (°C)	DTG (°C)	Residual Mass (%)
rPET	418.3	432.5	12.39
rPET/rGO-0.5	413.0	436.1	15.46
rPET/rGO-0.75	406.4	433.0	16.65
rPET/rGO-1.0	403.4	430.1	17.32
rPET/rGO-1.25	417.6	431.2	18.51

**Table 6:** Mean tensile strength, strain, and modulus values for rPET and rPET/rGO nanocomposites.

Sample	Tensile Strength (MPa)	Stretching (%)	Young Modulus (MPa)
rPET	2.9 ± 0.5	119.0 ± 29.0	27.7 ± 0.20
rPET/rGO-0.5	2.7 ± 0.2	71.0 ± 8.0	29.9 ± 0.05
rPET/rGO-0.75	1.8 ± 0.0	120.0 ± 7.0	16.7 ± 0.50
rPET/rGO-1.0	1.9 ± 0.4	44.0 ± 18.0	23.7 ± 1.80
rPET/rGO-1.25	1.2 ± 0.1	75.0 ± 19.0	9.9 ± 0.20

**Table 7:** Current stimuli values for Ag/AgCl and rPET/rGO-1.0 electrodes at frequencies of 5 and 3000 Hz.

		Ag/AgCl	I	II
Frequency (Hz)	Stimulated Limb	Current stimuli (µA)		
5	Right arm	177.6 ± 8.0	422.1 ± 20.1	339.4 ± 13.0
	Left arm	125.3 ± 6.2	279.9 ± 10.9	297.1 ± 10.3
3000	Right arm	1133.1 ± 53.6	1234.6 ± 58.7	1370.0 ± 63.4
	Left arm	1170.9 ± 53.5	1370.0 ± 65.3	1405.5 ± 50.7
