# Correction: Barbosa et al. Production of rGO-Based Electrospinning Nanocomposites Incorporated in Recycled PET as an Alternative Dry Electrode. *Polymers* 2022, *14*, 4288

**DOI:** 10.3390/polym17152056

**Published:** 2025-07-28

**Authors:** Michelle Chizzolini Barbosa, Claudia do Amaral Razzino, Thiago Domingues Stocco, Moisés das Virgens Santana, Anupama Ghosh, Luiz Fernando Pereira, Carlos Julio Tierra-Criollo, Anderson Oliveira Lobo

**Affiliations:** 1 Research and Development Institute, University of Vale do Paraiba—UNIVAP, São Jose dos Campos 12244-000, SP, Brazil; mi.chizzolinib@gmail.com (M.C.B.); claudiarazzino@gmail.com (C.d.A.R.); 2 Bioengineering Program, Scientific and Technological Institute, Brasil University, São Paulo 08230-030, SP, Brazil; thiago.stocco@ub.edu.br; 3 Interdisciplinary Laboratory for Advanced Materials, Materials Science and Engineering Graduate Program, Federal University of Piaui, Teresina 64049-550, PI, Brazil; moisesdvs@outlook.com; 4 Department of Chemical and Materials Engineering—DEQM, Pontifical Catholic University of Rio de Janeiro, Rio de Janeiro 22453-900, RJ, Brazil; anupama1984@gmail.com; 5 Biomedical Engineering Program-PEB, Federal University of Rio de Janeiro, Rio de Janeiro 21941-914, RJ, Brazil; f.pereiraluiz@peb.ufrj.br

During the final review of our manuscript [1], we identified an error in Figure 6, where panels 6a and 6b were inadvertently duplicated. The correct version of Figure 6a has now been prepared and is provided for replacement. This revision does not affect the results, conclusions, or interpretation of the study. This correction was approved by the Academic Editor. The original publication has also been updated.

## Figures and Tables

**Figure 6:**
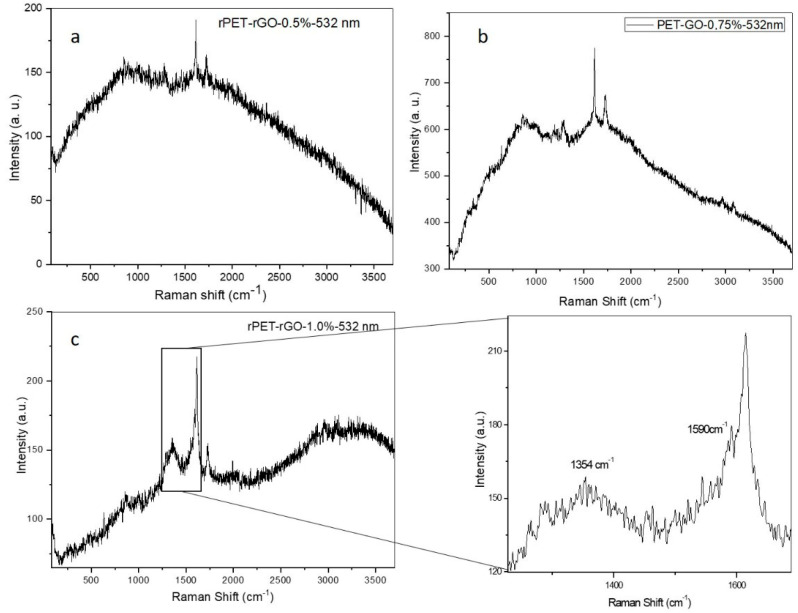
Raman spectra of (**a**) rPET/rGO-0.5; (**b**) rPET/rGO-0.75; and (**c**) rPET/rGO-1.0 composites, with an emphasis on the D and G bands, characteristic of graphene.
